# High Performance Gas Separation Mixed Matrix Membrane Fabricated by Incorporation of Functionalized Submicrometer-Sized Metal-Organic Framework

**DOI:** 10.3390/ma11081421

**Published:** 2018-08-13

**Authors:** Baosheng Ge, Yanyan Xu, Haoru Zhao, Haixiang Sun, Yaoli Guo, Wenguang Wang

**Affiliations:** 1 State Key Laboratory of Heavy Oil Processing, China University of Petroleum (East China), Qingdao 266580, China; gebaosheng@upc.edu.cn (B.G.); s16030434@s.upc.edu.cn (Y.G.); 2 College of Science, China University of Petroleum (East China), Qingdao 266580, China; 18266482821@163.com (Y.X.); z16090700@s.upc.edu.cn (H.Z.); z16090701@s.upc.edu.cn (W.W.); 3 Guangzhou Special Pressure Equipment Inspection and Research Institute, Guangzhou 510663, China

**Keywords:** mixed matrix membranes, metal–organic framework, gas separation, amination, submicron

## Abstract

Mixed matrix membranes (MMMs) attract great attention due to their outstanding gas separation performance. The compatibility between the fillers and the polymer matrix is one of the key points for the preparation of high-performance MMMs. In this work, MMMs consisting of metal-organic frameworks (MOFs) of amine-modified Cu-BTC (NH_2_-Cu-BTC; BTC = 1,3,5-benzenetricarboxylic acid) and submicrometer-sized amine-modified Cu-BTC (sub-NH_2_-Cu-BTC) incorporated into a Pebax-1657 polymer were fabricated for the gas separation. The SEM image and Fourier transform infrared spectroscopy (FTIR) spectra showed an increase in the surface roughness of MOFs and the presence of amino groups on the surface of Cu-BTC after the amination modification, and a decrease in the size of MOFs crystals after the submicrometer-sized aminated modification. Gas adsorption analysis indicated that NH_2_-Cu-BTC and sub-NH_2_-Cu-BTC had a higher gas adsorption capacity for CO_2_ compared to the unmodified Cu-BTC. The scanning electron microscopy (SEM) image showed that NH_2_-Cu-BTC and sub-NH_2_-Cu-BTC, especially sub-NH_2_-Cu-BTC, had a better compatibility with a polyether-block-amide (Pebax) matrix in the MMMs. The gas separation performance indicated that the Pebax/sub-NH_2_-Cu-BTC MMMs evidently improved the CO_2_/N_2_ and CO_2_/CH_4_ selectivity at the expense of a slight CO_2_ permeability. The results reveal that modified MOF-filled MMMs possess great potential for applications in the CO_2_ separation field.

## 1. Introduction

Carbon dioxide (CO_2_) is one of the main gases which cause global warming, corrode the natural gas pipelines, and reduce the combustion efficiency of natural gas. To overcome these problems, the effective separation of CO_2_/N_2_ and CO_2_/CH_4_ has become a hot research topic in recent years. Compared to other separation technologies such as cryogenic techniques, chemical adsorption [1], physical adsorption [2], and adsorption–separation [3], membrane separation has evolved as an important CO_2_ separation method owing to its low energy consumption, simple operation, low cost, and the absence of a phase transition during the separation process [4,5,6]. Polymer membranes are regarded as an effective media for the separation of gaseous mixtures accounting for a high separation efficiency, low running costs, and simple operating procedures. Nevertheless, the traditional polymer membranes for the gas separation have a near-universal trade-off phenomenon between the permeability and selectivity, as demonstrated by Robeson [7]. 

Mixed matrix membranes (MMMs) consisting of a polymer as the continuous phase and an inorganic filler as the dispersed phase can overcome Robeson’s upper-bound trade-off limit. The fillers embedded in the polymer matrix are classified into conventional fillers (zeolites [8], silicas [9], and metal oxides) and alternative fillers (carbon nanotubes (CNTs), metal–organic frameworks (MOFs), and graphene [8,10,11,12,13,14,15,16,17,18]). These fillers may lead to the development of high-performance membranes based on the properties and functionalization of fillers. However, still many problems should be solved for large-scale industrial production of MMMs. One of the most important problems is the generation of non-selective voids because of the incompatibility between the fillers and polymer matrix at the interface, which decreases the overall gas separation efficiency of the membrane.

MOFs are highly attractive for applications in the gas separation membranes because of high surface areas, large porosity, and high functionality compared to other fillers [19,20,21]. Moreover, the organic linkers in MOFs have a better affinity for the polymer chains compared to other fillers, and the surface properties of MOFs can be easily regulated by functionalization with various organic molecules [22]. The addition of MOFs in mixed matrix membranes can effectively control the interface morphology between the fillers and polymer matrix; this decreases the occurrence of defective morphologies [23]. However, there were still some nonselective voids present at the interface of MOFs and polymers attributed to the artifacts created during the sample preparation [24], which were unfavorable for the gas separation. To improve the MOF–polymer interfacial interactions, effective MOF modification techniques have been developed in the past few years, including the use of silane coupling agents [25] and particle fusion [26]. However, it is still challenging to achieve the desired interfacial morphology and improve the gas separation properties of MMMs. Synthesis of amine-modified and submicrometer-sized MOF crystals provides effective approaches to solve these problems [27,28,29]. 

Besides the fillers, it is equally important to select a suitable polymer for enhancing the separation properties of prepared membranes. Polyether-block-amide (Pebax) as a commercial thermoplastic elastomer is known for its selective penetration of CO_2_ over other light gases such as H_2_, N_2_, and CH_4_ based on the interactions between ethylene oxide (EO) units and CO_2_ [30]. Furthermore, Pebax is composed of polyoxyalkylene glycols (such as poly(ethylene oxide) (PEO) and poly(tetramethylene oxide) (PTMO)) and dicarboxylic acid terminated aliphatic polyamides (such as nylon-6 (PA6) and nylon-12 (PA12)). It has been known that the hard polyamide segment is favorable to improve the mechanical properties of the membranes, and the flexible polyether segment provides the passageway for the gas to permeate [31,32], therefore, different grades of Pebax can be obtained by varying the type and content of the segment. 

In this study, Cu-BTC crystals were fabricated using the hydrothermal method. Then amine-modified Cu-BTC (NH_2_-Cu-BTC) was prepared using the replacing ligands, and submicrometer-sized amine-modified Cu-BTC (sub-NH_2_-Cu-BTC) was prepared using the “coordination modulation method” (with sodium formate as the modulator). Three kinds of MOFs and Pebax-1657 were employed as the dispersed phase and the continuous phase, respectively, to prepare Pebax/Cu-BTC, Pebax/NH_2_-Cu-BTC, and Pebax/sub-NH_2_-Cu-BTC MMMs using the solution-casting method. It was expected that the dispersion of NH_2_-Cu-BTC and sub-NH_2_-Cu-BTC would be more homogeneous by amino functionalization, and the size of the crystals would be reduced. The chemical structure and morphology of the MOFs were confirmed by scanning electron microscopy (SEM), Fourier transform infrared spectroscopy (FTIR), and X-ray diffraction (XRD) analysis. The aggregate morphology of MOFs in a Pebax matrix was studied by SEM analysis. The permeability of pure gases in a Pebax membrane and Pebax/MOFs MMMs was determined by the constant volume–variable pressure approach. The corresponding separation mechanism of MOFs in MMMs for the gas separation was also discussed. Moreover, the effects of functionalized Cu-BTC on the solubility and diffusivity selectivity were systematically investigated.

## 2. Materials and Methods 

### 2.1. Materials

Pebax-1657 was purchased from Arkema Inc. (Paris, France). The main ligands 1,3,5-benzenetricarboxylic acid (BTC) and 2-aminoterephthalic acid (NH_2_-BDC) with 98% purity were supplied by Aladdin (Shanghai, China) and Macklin (Shanghai, China), respectively. Copper (II) nitrate trihydrate (Cu(NO_3_)_2_·3H_2_O) and sodium formate (HCOONa) were purchased from Sinopharm Chemical Reagent Co., Ltd. (Shanghai, China). Ethanol and *N*,*N*-dimethylformamide (DMF) were purchased from Sinopharm Chemical Reagent Co., Ltd. (Shanghai, China) and used as solvents without any purification.

### 2.2. Synthesis of MOFs

Cu-BTC was synthesized by adding 2.4 g Cu(NO_3_)_2_·3H_2_O, 1.2 g BTC, 20 mL bi-distilled water, 20 mL ethanol, and 20 mL DMF in a beaker, followed by stirring for 30 min and heating in a Teflon-lined steel autoclave at 85 °C for 20 h. Then, the obtained crystals were washed with DMF and first dried at 60 °C overnight and then dried at 120 °C for a day. NH_2_-Cu-BTC was synthesized in a similar manner as Cu-BTC except by replacing 25 wt.% BTC ligands with NH_2_-BDC in the initial synthesis compound using a mixture of 0.3 g NH_2_-BDC and 0.9 g BTC as the ligands. Sub-NH_2_-Cu-BTC was synthesized in a similar manner as NH_2_-Cu-BTC except by adding 0.1 g sodium formate to the reaction mixture.

### 2.3. Fabrication of MMMs

Pristine Pebax membrane and Pebax/MOFs MMMs were prepared using the conventional solution-casting method. To prepare a 4 wt.% solution of Pebax, 1.61 g Pebax pellets were dissolved in a mixture of 70 wt.% ethanol and 30 wt.% water under reflux at 80 °C for 2 h. Then, various quantities of MOFs (0, 1, 2, 3 and 4 wt.%) treated with different processes were sonicated in the Pebax solution described above for 30 min. The mixture was stirred for an additional 2 h at room temperature to obtain homogeneous dispersions containing different amounts of Cu-BTC, NH_2_-Cu-BTC, and sub-NH_2_-Cu-BTC. Then, the solutions were degassed by keeping them at room temperature for 10 h and cast on a flat glass plate. The obtained membranes were air-dried at ambient temperature for 24 h to complete the solvent evaporation. Finally, the membranes were placed in a vacuum oven for an additional 24 h to further ensure the complete removal of the residual solvent. The thickness of all MMMs was approximately 15–20 μm.

### 2.4. Characterization

The morphology of both MOF particles and MMMs was observed using a scanning electron microscope (SEM, S-4800, Hitachi, Tokyo, Japan). The samples were each coated with gold before the SEM measurements. To observe the cross-sections, the MMMs were cryogenically fractured in liquid nitrogen prior to coating with gold. The FTIR spectra of pristine and modified Cu-BTC were obtained using a BRUKER TENSORII FT-IR spectrometer (Billerica, MA, USA) in the wavenumber range 4000−600 cm^−1^. The XRD spectra of pristine and functionalized Cu-BTC, pristine Pebax membrane, and the MMMs incorporated with MOFs were obtained using a PANalytical X’Pert PRO Materials Research diffractometer (Almelo, The Netherlands) using a voltage of 40 kV and a current of 40 mA. The X-rays (λ = 1.5418 Å) were generated from a Cu Kα source. Each pattern was collected in the 2*θ* range 5–40° in the repetition mode (three times) with a total duration of approximately 0.4 h at selected times of hydration. To investigate the thermal stability of MOFs, thermogravimetric analysis (TGA) was carried out under air flow using a TA instrument (NETZSCH STA 449F5, Selb, Germany). All MOFs were heated from 25 °C to 1000 °C at a heating rate of 10 °C/min. The adsorption capacity of CO_2_, N_2_, and CH_4_ for the MOFs were measured by ASAP-2020 gas adsorption tester produced by Ningbo Oppe Instruments Co., Ltd. (Ningbo, China). The crystals were immersed in dichloromethane and methanol for three days (the solvents were changed once a day), then filtered for 5 h at 60 °C. Then the adsorption amount of CO_2_, N_2_, and CH_4_ of the MOFs was measured at 308 K. The nitrogen adsorption of the MOFs was characterized by ASAP2020M specific surface area and a micropore physical adsorption analyzer produced by Micromeritics Company (Atlanta, GA, USA) at 77 K, with a sample quantity of 0.617 g. Considering the influence of MOFs’ gas adsorption capacity on the selective CO_2_ permeability performance of MMMs, the gas uptake of pristine and functionalized Cu-BTC was also measured. Before the measurement, the as-synthesized crystal samples were immersed in methanol to exchange the uncoordinated DMF, ethanol, and water molecules. Then, the samples were dried at 60 °C under high vacuum for 3 h to obtain the activated samples.

### 2.5. Procedure for Gas Permeability Measurements

Pure gas (CO_2_, N_2_, and CH_4_) permeability measurements were carried out using a pressure permeability tester (Labthink Instruments, Jinan, China). The experiments were performed at 25 °C. A circular membrane with an effective area of 4.95 cm^2^ was mounted in a permeation cell prior to degassing the entire apparatus. Gas permeability coefficients (*P*) can be calculated as follows [33]:

(1) $$P=\frac{273}{76}\times \frac{VL}{ATp_{0}}\times \frac{dp}{dt}$$

where *P* is the permeability of a membrane to a gas, and its unit is Barrer (1 Barrer = 1 × 10^−10^ cm^3^ cm cm^−2^ s^−1^ cm Hg^−1^); *V* is the volume of the down-stream chamber (cm^3^); *L* and *A* are the thickness (cm) and effective area of the membrane (cm^2^), respectively; *T* is the experimental temperature (K); *p*_0_ is the pressure in cmHg of the gas in the upstream chamber, and *dp*/*dt* is the rate of pressure increase at the low-pressure chamber (the permeate side). The absolute temperature of 0 °C and standard atmospheric pressure are 273 K and 76 cm Hg, respectively. For the permeation measurement, the values were repeated at least three times to verify the reproducibility, and the relative standard deviation was within 5%.

The apparent diffusion coefficient (*D*) was obtained from the Equation (2):

(2) $$D=\frac{L^{2}}{6\theta }$$

where *θ* is the lag time when a steady *dp*/*dt* rate is obtained on the downstream side. 

The ideal selectivity *α_A/B_* can be defined as follows:

(3) $$\alpha_{A/B}=\frac{P_{A}}{P_{B}}$$

where *P_A_* and *P_B_* represent the permeability of gases A and B, respectively. Here, *P_A_* and *P_B_* are the permeability of pure gas CO_2_ and N_2_, or CO_2_ and CH_4_ (Barrer), respectively.

## 3. Results and Discussion

### 3.1. Characterization of MOFs

#### 3.1.1. SEM

**Figure 1:**
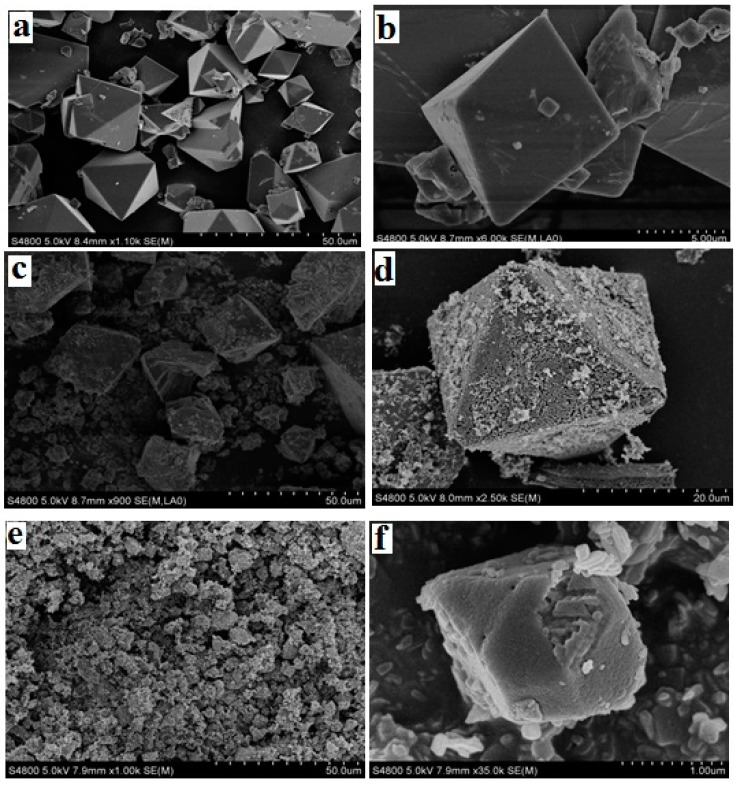
Scanning electron microscope (SEM) images of the surface of (**a**,**b**) pristine Cu-BTC, (**c**,**d**) NH_2_-Cu-BTC and (**e**,**f**) sub-NH_2_-Cu-BTC. Note BTC = 1,3,5-benzenetricarboxylic acid.

The morphological features of the pristine and functionalized MOFs were investigated by SEM, shown in Figure 1. The pristine Cu-BTC is in the form of cube-shaped crystals [23] with a particle size of ~18 μm. After the amino functionalization, the crystal size and shape of NH_2_-Cu-BTC displays no substantial change compared to the Cu-BTC crystal, whereas some whisker-like roughness is observed on the crystal surface [27]. The roughness of the crystal surface is mainly due to the replacement of a part of the BTC ligand with NH_2_-BDC, and the addition of NH_2_-BDC leads to the secondary nucleation on the crystal surface, which causes the crystal surface become rougher. With the addition of a certain amount of sodium formate, the size of the sub-NH_2_-Cu-BTC crystals leads to a decrease from the original size of 18 to 2 μm, whereas the shape and surface texture of crystals has no apparent variation.

#### 3.1.2. FTIR

**Figure 2:**
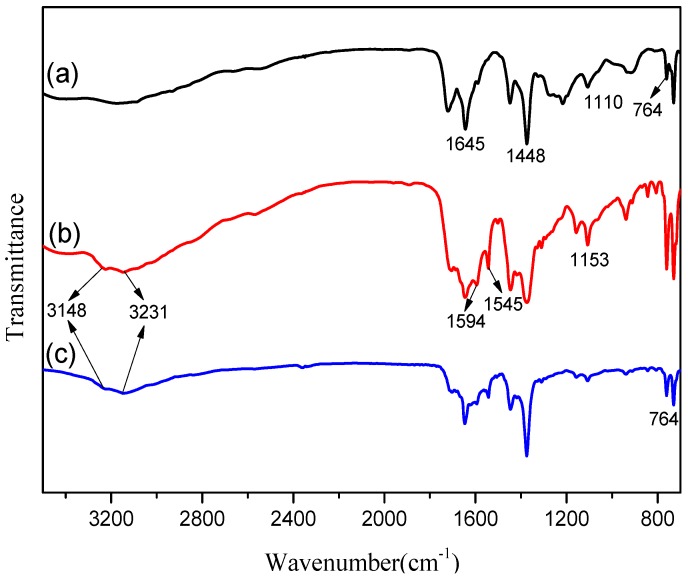
Fourier transform infrared spectroscopy (FTIR) spectra of (**a**) Cu-BTC, (**b**) NH_2_-Cu-BTC and (**c**) sub-NH_2_-Cu-BTC.

The FTIR experiment was conducted to determine the functional groups present on the surface of the MOFs. Figure 2 shows the FTIR spectra of pristine Cu-BTC, NH_2_-Cu-BTC, and sub-NH_2_-Cu-BTC powders in the wavenumber range of 3500–700 cm^−1^. For the pristine Cu-BTC, the peaks at the wavenumber 1645 cm^−1^ and 1448 cm^−1^ correspond to the C-C skeletal vibration of benzene groups in the BTC linker and the asymmetric COO stretching, respectively [34]. The peak at 1110 cm^−1^ corresponds to C-O-Cu stretching [35], and the band around 764 cm^−1^ represents Cu substitution on benzene groups. The new peak appearance at 1594 cm^−1^ in the NH_2_-Cu-BTC crystal can be attributed to the asymmetric COO band of the carboxylate group of BDC in the presence of DMF [36], confirming the presence of BDC linker in NH_2_-MOF. The middle peak at 1545 cm^−1^ is representative of the C-N bond, and the peak intensity indicates the existence of primary amine and no secondary amine. In addition, a peak appears at 1153 cm^−1^ in correspondence to the stretching of C-N bond, and the two peaks at 3148 cm^−1^ and 3231 cm^−1^ are attributed to N-H, further proving the existence of amino groups in NH_2_-Cu-BTC. For the sub-NH_2_-Cu-BTC powder shown in (**c**) of Figure 2, the FTIR spectrum is almost the same as that of NH_2_-Cu-BTC powder, which indicates that the modified submicrometer method only reduces the crystal size without changing the functional groups on the crystal surface.

#### 3.1.3. XRD

**Figure 3:**
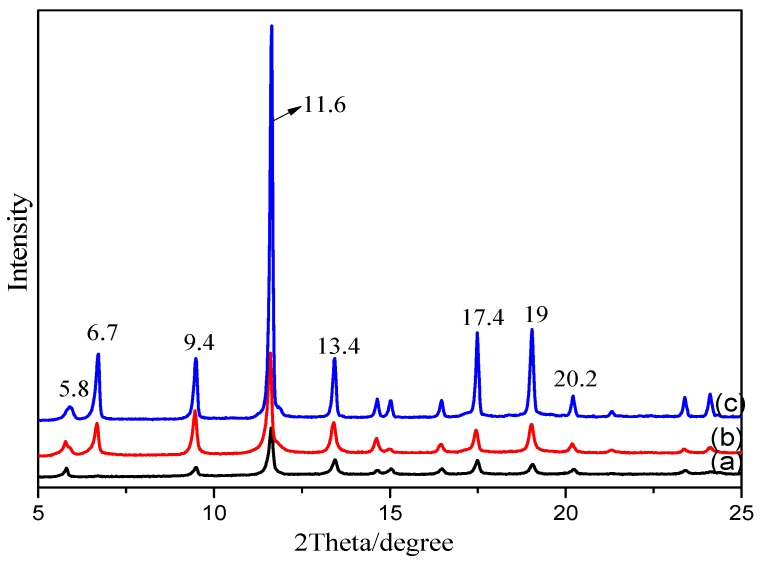
The X-ray diffraction (XRD) patterns of (**a**) Cu-BTC, (**b**) NH_2_-Cu-BTC and (**c**) sub-NH_2_-Cu-BTC.

To investigate the effects of different modification methods on the crystal structure of MOFs, the as-synthesized MOFs were characterized by XRD. Figure 3 indicates that the three types of MOFs have highly crystalline structures. The sharp peaks at 2*θ* 5.8, 6.7, 9.4, 11.6, 13.4, 17.4, and 19.0° confirm the ordered crystalline structure of Cu-BTC, which is consistent with the reported XRD spectra of MOFs in the literature [27]. The addition of a new ligand containing an –NH_2_ functional group in the spectra of the NH_2_-Cu-BTC and sub-NH_2_-Cu-BTC has no obvious effect on the XRD spectra, which indicates that the modification does not affect the crystal structure of the MOFs. After the functionalization modification, the intensity of Cu-BTC significantly increased, indicating that the crystallinity of the MOFs has been improved. The reason for the increase of the peak intensity may be due to the increase of crystallinity caused by the two nucleation process after the addition of NH_2_-BDC as ligands.

#### 3.1.4. Thermal Properties

**Figure 4:**
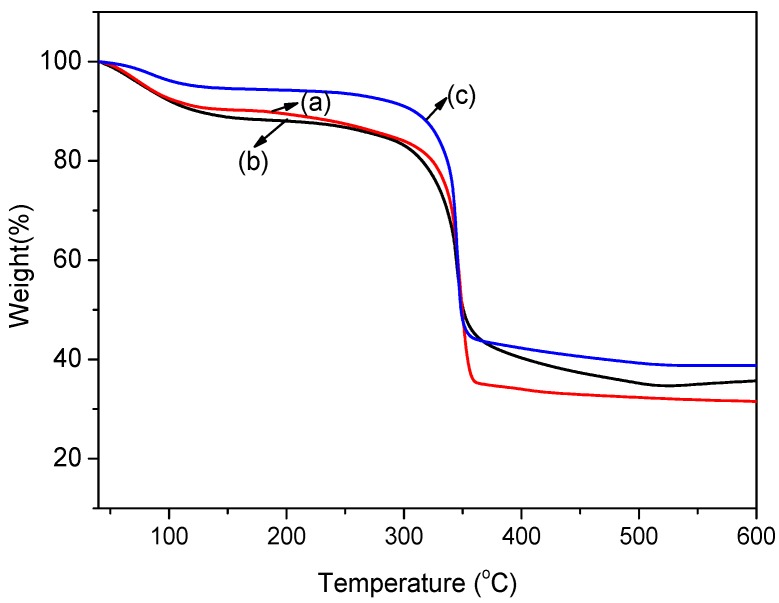
Thermogravimetric analysis (TGA) curves of (**a**) Cu-BTC, (**b**) NH_2_-Cu-BTC and (**c**) sub-NH_2_-Cu-BTC.

TGA is an important method to investigate the effect of different modification methods on the thermal stability of MOFs. The weight loss curves of the as-prepared MOFs are shown in Figure 4. The weight loss curve of pristine Cu-BTC and NH_2_-Cu-BTC showed that about 10 wt.% of the material was lost at 100 °C. This can be attributed to the trapped solvent in the pores and the presence of water on the surface of the crystals. The crystals remain stable up to 300 °C, as reported earlier [37], and above this temperature, the crystals start to decompose. Submicrometer-sized NH_2_-Cu-BTC crystals follow a similar trend except that the weight loss at 100 °C is slightly smaller. The reason for this is that the amount of residual solvent in the sub-NH_2_-Cu-BTC crystals is lower than that of the Cu-BTC and NH_2_-Cu-BTC due to the smaller pore volume of crystals. 

#### 3.1.5. Gas Adsorption Measurements

**Figure 5:**
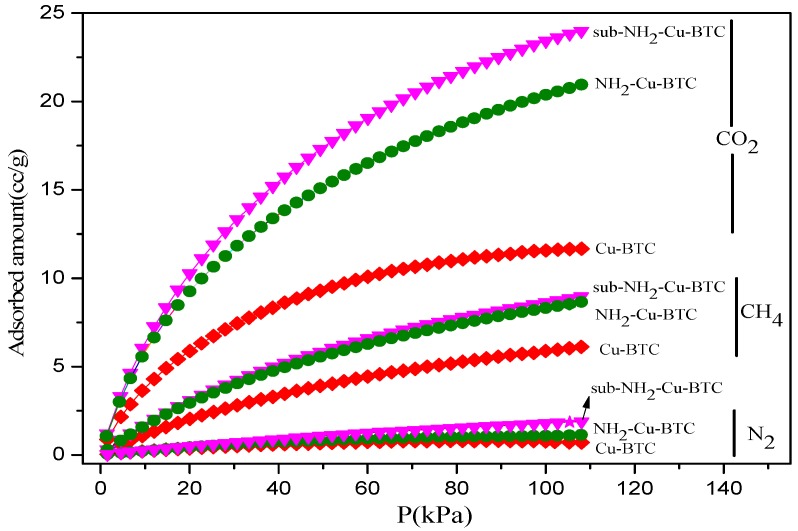
CO_2_, N_2_, and CH_4_ adsorption isotherms of Cu-BTC at 308 K.

Figure 5 shows the CO_2_, N_2_, and CH_4_ adsorption isotherms of the MOFs at 308 K, and Table 1 shows the physical and textural properties of pristine and functionalized Cu-BTC including the Brunauer-Emmett-Teller (BET) surface area, Langmuir surface area, pore volume, and the maximum adsorption amounts of CO_2_, CH_4_, and N_2_. According to the results of the N_2_ adsorption isotherms at 77 K, the amount of N_2_ adsorbed by Cu-BTC is significantly higher than that absorbed by NH_2_-Cu-BTC and sub-NH_2_-Cu-BTC. This indicates that Cu-BTC has a higher BET surface area and micropore volume, as shown in Table 1. The CO_2_ uptake of three types of MOFs significantly increases with the increase in adsorption pressure owing to the strong interactions between the positive charges on the unsaturated open Cu metal and the quadrupolar CO_2_ molecules. The amount of CO_2_ adsorbed per unit mass is higher than that of N_2_ and CH_4_, and the amounts of CO_2_ adsorbed by sub-NH_2_-Cu-BTC and NH_2_-Cu-BTC are 23.99 cc/g and 21.01 cc/g, respectively, higher than the amount of CO_2_ absorbed by Cu-BTC (11.62 cc/g). This behavior is inconsistent with the BET results shown in Table 1, suggesting that the micropore volume of NH_2_-Cu-BTC and sub-NH_2_-Cu-BTC reduced compared to Cu-BTC due to the partial substitution of BTC linker with NH_2_-BDC. This can be attributed to the presence of primary amino groups in the porous structure, creating an electric field inside the pores against more polarizable adsorbates. In addition, the free primary amine present in NH_2_-Cu-BTC formed a carbamate with the CO_2_, providing a high energy efficiency [38], which is favorable to improve the CO_2_ separation. The increase of N_2_ and CH_4_ adsorption capacity is due to the increase of the surface roughness after the amine functionalization.

### 3.2. Characterization of MMMs

#### 3.2.1. SEM

**Figure 6:**
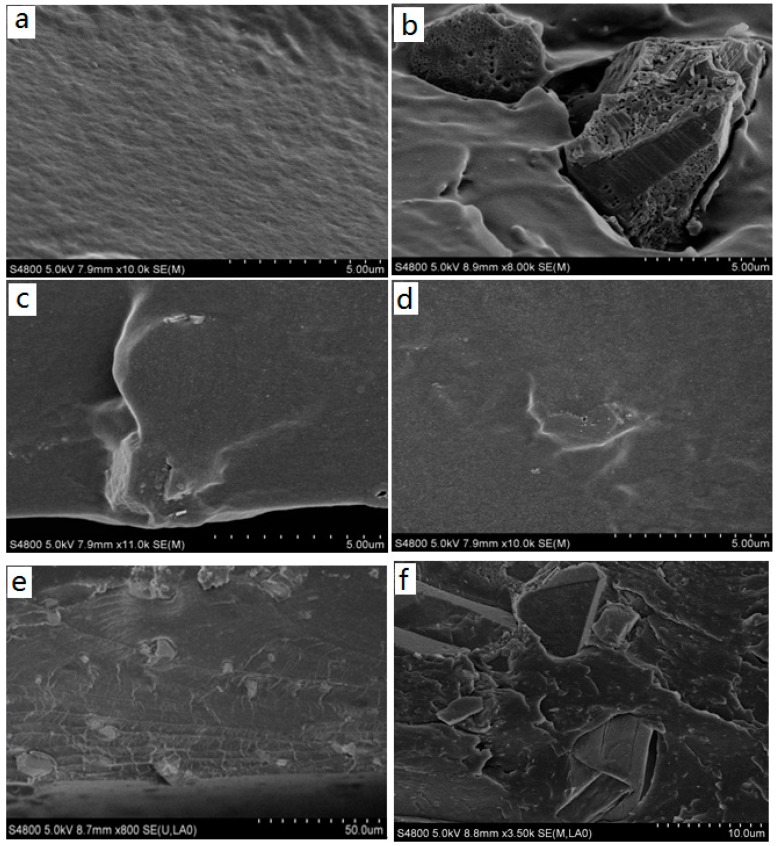
The SEM images of cross-section of (**a**) polyether-block-amide (Pebax) membrane, (**b**) Pebax/Cu-BTC mixed matrix membranes (MMMs) (3 wt.% MOF content in the MMMs), (**c**) Pebax/NH_2_-Cu-BTC MMMs (3wt.% MOF content in the MMMs), (**d**) Pebax/sub-NH_2_-Cu-BTC MMMs (3 wt.% metal-organic framework (MOF) content in the MMMs), (**e**) the low magnification of (**d**) and (**f**) Pebax/sub-NH_2_-Cu-BTC MMMs (4 wt.% MOF content in the MMMs).

The dispersion of MOF particles within the polymer matrix and the internal microstructure of MMMs were characterized from the cross-section SEM images shown in Figure 6. It can clearly be seen that the Pebax membrane is smooth and defect-free. The introduction of Cu-BTC has a significant effect on the membrane morphology. The void spaces emerge between the Cu-BTC particles and polymeric matrix based on the poor distribution of the unmodified Cu-BTC particles in the polymer matrix and have poor compatibility with the polymer matrix. The cross-section SEM images of the MMMs incorporated with NH_2_-Cu-BTC and sub-NH_2_-Cu-BTC show that the MOFs inside the polymer matrix are defect-free and have good compatibility. A better adhesion of the NH_2_-Cu-BTC surface with the Pebax matrix can be attributed to the formation of hydrogen bonding between the amine groups of NH_2_-Cu-BTC and the hydroxyl groups of Pebax. This indicates that the two modification methods improved the interface compatibility between the Pebax matrix and filler particles. However, the MOFs agglomerations and clusters are clearly observed in (**f**) of Figure 6 when 4 wt.% sub-NH_2_-Cu-BTC is incorporated into the Pebax matrix, indicating that the MOF is poorly dispersed in the polymer at higher loadings. This aggregation of MOFs would increase the void spaces between the polymer matrix and nanoparticles, which improves the CO_2_ and N_2_ permeability at the same time, leading to the deterioration of the gas separation selectivity.

#### 3.2.2. XRD

**Figure 7:**
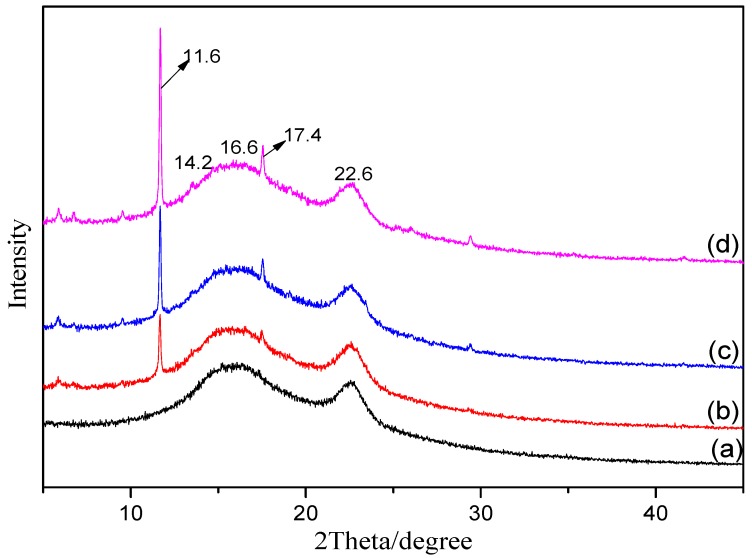
The XRD patterns of (**a**) pure Pebax membrane, (**b**) Pebax/Cu-BTC MMMs, (**c**) Pebax/NH_2_-Cu-BTC MMMs and (**d**) Pebax/sub-NH_2_-Cu-BTC MMMs (3 wt.% MOFs content in the MMMs).

Figure 7 shows the XRD patterns of pure Pebax and Pebax/MOFs membranes between 5° and 35° to determine their crystalline structure. The pristine Pebax membrane shows a broad peak at 16.6° of 2*θ* and a narrow diffraction peak at 22.6°, suggesting the semicrystalline structure of the sample [39] similar to the reported XRD pattern for the Pebax membrane in the literature [40]. In addition, the peak at 2*θ* = 22.6° can be attributed to the crystalline region of polyamide (PA) segment formed by hydrogen bonding [41]. For the diffraction patterns of Pebax/MOF membranes, the intensity and position of characteristic peaks of the Pebax membrane are almost constant with the incorporation of MOFs, indicating that the chain packing and intersegmental spacing of the Pebax polymer had no substantial change. Two new characteristic peaks of MOFs at 2*θ* = 11.6° and 17.4°, corresponding to (222) and (422), are observed at a low loading. Furthermore, Figure 7 also shows that the intensity of the two characteristic peaks of MMMs doped with NH_2_-Cu-BTC and sub-NH_2_-Cu-BTC at 2*θ* = 16.6° and 22.6° slightly decreased compared to those doped with Cu-BTC. This is because the polymer crystallinity decreased due to the formation of hydrogen bonds caused by the interactions between the amine functional groups on the surface of NH_2_-Cu-BTC and sub-NH_2_-Cu-BTC with the PEO component of Pebax.

### 3.3. Gas Separation Performances

**Figure 8:**
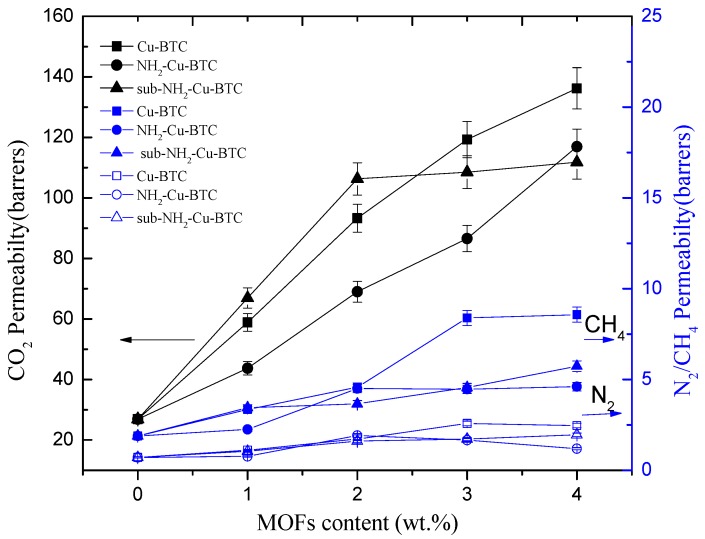
Effects of the MOFs loading level on the gas permeability of MMMs.

**Figure 9:**
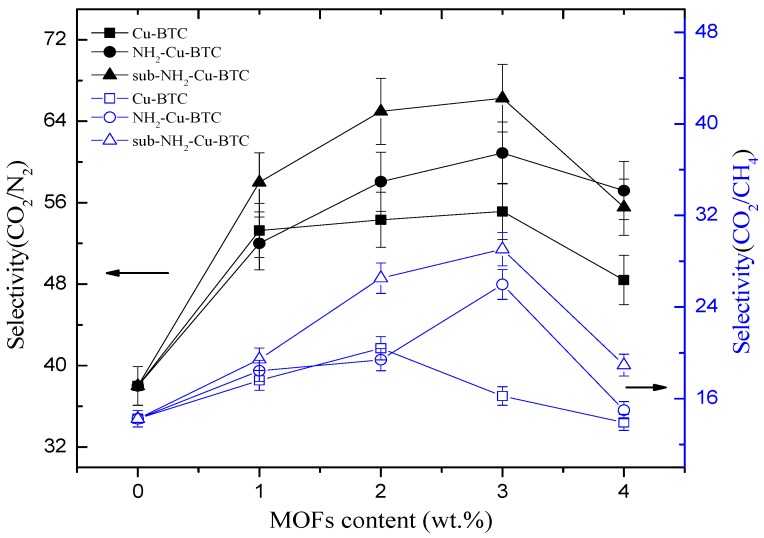
Effects of MOFs loading level on the gas separation selectivity.

The gas permeation properties of MMMs are mainly affected by the interface morphology of the continuous and dispersed phases. To investigate the effect of different types of MOFs and filler loadings on the membrane separation properties, Pebax/MOFs MMMs with different loadings of Cu-BTC, NH_2_-Cu-BTC, or sub-NH_2_-Cu-BTC were fabricated. The gas permeability and selectivity of CO_2_/N_2_ and CO_2_/CH_4_ at 25 °C and 0.1 MPa of the feed pressure are shown in Figure 8 and Figure 9, respectively. All experiments were performed at least three times, and the average results are reported. A comparable increase in the CO_2_ permeability of MMMs fabricated with different types of MOFs with the Pebax membrane results in an increase in the selectivity of CO_2_/N_2_ and CO_2_/CH_4_ than that of the pristine Pebax membrane. The CO_2_ permeability of MMMs increased with the increase of Cu-BTC loading, and the improved permeability can be mainly attributed to the defects at the inorganic–polymer interface. Moreover, the strong quadrupole moment of CO_2_ has a higher affinity with unsaturated Cu sites than N_2_ and CH_4_, but the increase in the N_2_ and CH_4_ permeability is not clear. The results are consistent with the results of N_2_, CO_2_, and CH_4_ adsorption measurements. Furthermore, the CH_4_ permeability is slightly higher than that of N_2_. This is probably because the critical temperature of gases decreases in the following order: CO_2_ (304.2 K) > CH_4_ (190.7 K) > N_2_ (126.1 K). A higher condensability shows a higher solubility of the gas in the polymer matrix [32], which results in a higher permeability. Therefore, the selectivity of CO_2_/N_2_ is higher than that of CO_2_/CH_4_. The selectivity of CO_2_/N_2_ and CO_2_/CH_4_ also increased with the increase in Cu-BTC loading. However, above the Cu-BTC loading of 4 wt.%, both the selectivity of CO_2_/N_2_ and CO_2_/CH_4_ of MMMs decreased due to poor compatibility and presence of nonselective voids at the inorganic-polymer interface.

Figure 9 and Table 2 show that the MMMs incorporated with NH_2_-Cu-BTC exhibit a higher selectivity of CO_2_/N_2_ and CO_2_/CH_4_ compared to that doped with pristine Cu-BTC at the expense of CO_2_ permeability. The decreased permeability can be attributed to the good compatibility between the amine functionalized fillers and Pebax [28]. The interaction between CO_2_ molecules and the amino groups on the surface of NH_2_-Cu-BTC and the decrease of nonselective voids increase the gas separation selectivity.

Furthermore, Figure 8 and Table 2 show that the CO_2_ permeability of MMMs incorporated with sub-NH_2_-Cu-BTC is higher than that of MMMs doped with NH_2_-Cu-BTC, but worse than that of pristine Cu-BTC. The presence of nanoparticles in the polymer matrix disrupts the chain packing and changes the structural regularity of the polymer–nanoparticle interface. Void spaces are formed due to weak compatibility at the interface of the polymer and nanoparticle phases that serve as channels to transport gas molecules more effectively. As shown in Figure 6, the MMMs incorporated with pristine Cu-BTC had worse compatibilities with the polymer matrix, which exhibited a higher CO_2_ permeability compared to the functionalized Cu-BTC. The permeability of MMMs doped with sub-NH_2_-Cu-BTC can be attributed to the smaller size of fillers after the submicrometer treatment and the presence of more amino and carboxyl groups on the crystal surface at the same loadings. Correspondingly, the MMMs incorporated with sub-NH_2_-Cu-BTC show a better selectivity of CO_2_/N_2_ and CO_2_/CH_4_. However, at a higher MOFs loading ratio, such as the loading above 4 wt.%, the increase in the rate of CH_4_ and N_2_ permeability is higher than that of the CO_2_ permeability based on the void spaces between the polymer matrix and nanoparticles, which leads to the deterioration of the gas separation selectivity. Compared with results reported in other literature, which directly incorporated pristine MOFs or amine functionalized MOFs [19,42], we found that the dispersion of functionalized MOFs became better in the Pebax matrix if the crystal size was effectively regulated. The CO_2_ permeability in the Pebax/sub-NH_2_-Cu-BTC was 303% higher than neat Pebax due to the fine dispersion and the presence of groups with a superior CO_2_-philicity in the framework. The ideal selectivity of CO_2_/N_2_ and CO_2_/CH_4_ was equally higher than that of the neat matrix.

According to the solution-diffusion model, gas permeation through a dense membrane depends on the diffusivity coefficient (*D*) and solubility coefficient (*S*). To further elucidate the role of fillers in gas permeation, the *D* value was calculated according to the Equation (2) and *S* values of the membranes were calculated according to the equation *S* = *P*/*D*. The diffusivity and solubility selectivities of CO_2_/N_2_ and CO_2_/CH_4_ of a pristine Pebax membrane and MMMs at 25 °C and 0.1 MPa are shown in Table 3. It can clearly be seen that both the CO_2_/N_2_ and CO_2_/CH_4_ diffusivity selectivities of these MMMs containing MOFs have no improvement compared to a pristine Pebax membrane. However, the solubility selectivities of CO_2_/N_2_ and CO_2_/CH_4_ are higher than those of a pristine Pebax membrane. Thus, the separation efficiency is based on the gas solubility, but not on the size differences. The unsaturated Cu sites and carboxyl groups of Cu-BTC enhance the affinity with CO_2_ molecules, and after the amination functionalization, the amide groups of the MOFs further increase the solubility selectivity of the MMMs due to the Lewis acid-base interactions with CO_2_ molecules. Besides, it is important to note that the introduction of primary amino groups on the MOFs leads to the formation of reactivity-selective membranes. Therefore, the extremely high CO_2_/N_2_ and CO_2_/CH_4_ selectivities of the MMMs can be attributed to the solubility selectivity. 

**Figure 10:**
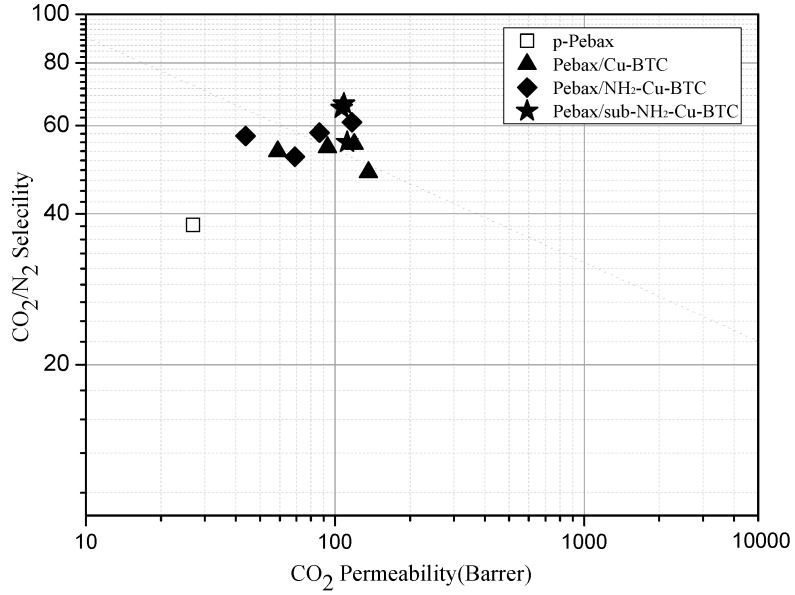
CO_2_/N_2_ separation performance of pure Pebax (□), Pebax/Cu-BTC (▲), Pebax/NH_2_-Cu-BTC (◆) and Pebax/sub-NH_2_-Cu-BTC (★) MMMs in Robeson upper bound plot (2008).

Figure 10 shows the trade-off lines between the selectivity and permeability for CO_2_/N_2_ separation (Robeson plot). The performance data of CO_2_ and N_2_ of a pristine Pebax membrane falls far below the updated upper bound reported in 2008 [7]. The Cu-BTC and NH_2_-Cu-BTC containing membranes are close to or surpass the upper bound plot, and the MMMs incorporated with sub-NH_2_-Cu-BTC clearly surpass that line. This demonstrates the promising performance of submicrometer-functionalized Cu-BTC compared to the untreated Cu-BTC in the CO_2_ separation field.

## 4. Conclusions

In this work, MMMs based on Pebax and Cu-BTC particles (including Cu-BTC, NH_2_-Cu-BTC, and sub-NH_2_-Cu-BTC) were fabricated using the solution-casting method. SEM analysis showed that the surface roughness of NH_2_-Cu-BTC clearly increased, and the size of sub-NH_2_-Cu-BTC clearly decreased. The resulting fine dispersion of MOFs throughout the Pebax matrix and a strong interfacial adhesion between the modified MOFs and the polymer matrix were obtained. FTIR analysis confirmed that the amine modification introduced amino functional groups (–NH_2_) on the surface of Cu-BTC. The XRD patterns obtained to establish the consistency of the crystal structure indicated that the modification process had no influence on the crystal structure of the MOFs. The order of different MOFs’ adsorption capacities of N_2_, CH_4_, and CO_2_ obtained by gas adsorption measurements at 308 K indicated that amine modification and submicrometer-sized amine modification were favorable for the increase of the gas adsorbability of MOFs. TGA showed that the crystals of three kinds of MOFs remained stable up to 300 °C. Gas separation performance showed that the MMMs with NH_2_-Cu-BTC and sub-NH_2_-Cu-BTC represented a better gas separation selectivity than that of MMMs incorporated with the pristine Cu-BTC, and the separation selectivity increased with the increase in MOF loading at low MOF contents. This can be attributed to the fine dispersion of NH_2_-Cu-BTC and sub-NH_2_-Cu-BTC and the presence of amino functional groups. However, the gas permeability of NH_2_-Cu-BTC and sub-NH_2_-Cu-BTC was slightly sacrificed because of the decrease in interfacial voids between the MOFs and the Pebax matrix. The promising performance of submicrometer-functionalized Cu-BTC in the fabrication of polymer membranes for effective CO_2_/N_2_ and CO_2_/CH_4_ separation was demonstrated.

## Figures and Tables

**Table 1:** Physical and textural properties of Cu-BTC.

Sample	S_BET_ (m^2^/g)	S_Langmuir_ (m^2^/g)	Pore Volume (m^3^/g)	CO_2_ Adsorption Amount (cc/g)	CH_4_ Adsorption Amount (cc/g)	N_2_ Adsorption Amount (cc/g)
Cu-BTC	1018	1191	0.48	11.62	6.05	0.67
NH_2_-Cu-BTC	797	847	0.39	21.01	8.61	1.11
sub-NH_2_-Cu-BTC	718	724	0.35	23.99	8.94	1.87

**Table 2:** Gas permeability and CO_2_/N_2_ and CO_2_/CH_4_ selectivity of the prepared membranes.

Type of Membrane	Permeability (Barrer)			Selectivity	
	N_2_	CH_4_	CO_2_	CO_2_/N_2_	CO_2_/CH_4_
Pebax	0.71	1.89	26.89	38.00	14.24
Pebax/Cu-BTC	2.16	7.35	119.3	55.13	16.23
Pebax/NH_2_-Cu-BTC	1.42	3.33	86.58	60.88	25.97
Pebax/sub-NH_2_-Cu-BTC	1.64	3.73	108.5	66.27	29.05

3 wt.% MOFs content in MMMs.

**Table 3:** Diffusivity and solubility selectivity of CO_2_/N_2_ and CO_2_/CH_4_.

Type of Membrane	D_CO2_/D_N2_	D_CO2/CH4_	S_CO2/N2_	S_CO2/CH4_
Pebax	2.01	3.13	19.02	4.55
Pebax/Cu-BTC	2.04	3.41	27.02	4.76
Pebax/NH_2_-Cu-BTC	1.98	3.77	30.75	6.89
Pebax/sub-NH_2_-Cu-BTC	2.01	3.92	32.97	7.41

3 wt.% MOFs Content in MMMs.
